# Proteomic Analysis of *Lactobacillus nagelii* in the Presence of *Saccharomyces cerevisiae* Isolated From Water Kefir and Comparison With *Lactobacillus hordei*

**DOI:** 10.3389/fmicb.2019.00325

**Published:** 2019-02-28

**Authors:** Julia Bechtner, Di Xu, Jürgen Behr, Christina Ludwig, Rudi F. Vogel

**Affiliations:** ^1^Lehrstuhl für Technische Mikrobiologie, Wissenschaftszentrum Weihenstephan, Technische Universität München, Freising, Germany; ^2^Bavarian Center for Biomolecular Mass Spectrometry, Freising, Germany

**Keywords:** *Lactobacillus nagelii*, *Lactobacillus hordei*, functional genome prediction, proteomic analysis, metabolism

## Abstract

Water kefir is a slightly alcoholic and traditionally fermented beverage, which is prepared from sucrose, water, kefir grains, and dried or fresh fruits (e.g., figs). *Lactobacillus* (*L.*) *nagelii, L. hordei*, and *Saccharomyces* (*S.*) *cerevisiae* are predominant and stable lactic acid bacteria and yeasts, respectively, isolated from water kefir consortia. The growth of *L. nagelii* and *L. hordei* are improved in the presence of *S. cerevisiae*. In this work we demonstrate that quantitative comparative proteomics enables the investigation of interactions between LAB and yeast to predict real-time metabolic exchange in water kefir. It revealed 73 differentially expressed (DE) in *L. nagelii* TMW 1.1827 in the presence of *S. cerevisiae*. The presence of the yeast induced changes in the changes in the carbohydrate metabolism of *L. nagelii* and affected reactions involved in NAD^+^/NADH homeostasis. Furthermore, the DE enzymes involved in amino acid biosynthesis or catabolism predict that *S. cerevisiae* releases glutamine, histidine, methionine, and arginine, which are subsequently used by *L. nagelii* to ensure its survival in the water kefir consortium. In co-culture with *S. cerevisiae, L. nagelii* profits from riboflavin, most likely secreted by the yeast. The reaction of *L. nagelii* to the presence of *S. cerevisiae* differs from that one of the previously studied *L. hordei*, which displays 233 differentially expressed proteins, changes in citrate metabolism and an antidromic strategy for NAD^+^/NADH homeostasis. So far, aggregation promotion factors, i.e., formation of a specific glucan and bifunctional enzymes were only detected in *L. hordei*.

## Introduction

Water kefir is a slightly alcoholic, traditionally fermented beverage, which is prepared from sucrose, water, kefir grains, and dried or fresh fruits (e.g., figs). Water kefirs, originating from definitely different sources, exhibit different species diversities. Still, the basic consortium, which mainly consists of lactic acid bacteria (LAB), acetic acid bacteria (AAB) and yeasts (Ward, 1891; Neve and Heller, 2002; Gulitz et al., 2011; Marsh et al., 2013; Laureys and De Vuyst, 2014) appears to be stable. *L. hordei, L. nagelii*, and *S. cerevisiae* are dominant LAB and yeast species, respectively, isolated from water kefir grains (Gulitz et al., 2011; Stadie et al., 2013; Laureys and De Vuyst, 2014). Among *L. hordei* and *L. nagelii* isolates from these water kefirs *L. hordei* TMW 1.1822 and *L. nagelii* 1.1827 were the most abundant isolates, which also produced dextrans and showed synergisms with concomitant yeasts (Stadie et al., 2013; Xu et al., 2018, 2019a).

In contrast to milk kefir, there is only very limited research on water kefir. Most of the available studies focused on its species diversity (Ward, 1891; Pidoux, 1989; Neve and Heller, 2002; Gulitz et al., 2011; Marsh et al., 2013; Laureys and De Vuyst, 2014; Martínez-Torres et al., 2017), or on the chemical and structural composition of the water kefir grains (Horisberger, 1969; Pidoux et al., 1988; Pidoux et al., 1990; Waldherr et al., 2010; Fels et al., 2018; Xu et al., 2018). To date, several attempts have been made to understand the interactions of the microorganisms in water kefir. For instance, Stadie et al. (2013) studied the metabolic interaction between LAB (*L. hordei* and *L. nagelii*) and yeasts (*S. cerevisiae* and *Zygotorulaspora florentina*) isolated from water kefir and inferred, that the growth of *L. hordei* TMW 1.1822 should be improved by nutrients produced by both yeasts, such as several amino acids (isoleucine, leucine, methionine, phenylalanine, tryptophan, tyrosine, and valine) and vitamin B6.

Another study explored the metabolite dynamics in a water kefir fermentation. The major metabolites produced were ethanol and lactic acid during 192 h of fermentation. Glycerol, acetic acid, and mannitol were produced in low concentrations. The prevailing volatile aroma compounds were ethyl acetate, isoamyl acetate, ethyl hexanoate, ethyl octanoate, and ethyl decanoate after 72 h (Laureys and De Vuyst, 2014). Further, the water kefirs were supplied with dried figs, apricots and raisins, respectively, as different nutrient sources delivering various concentrations. Also, the influence of oxygen has been investigated. It was concluded, that raisins led to low nutrient concentrations in the water kefir formulation, which favored the growth of *L. hilgardii* and *Dekkera bruxellensis*. In contrast, figs supplied the water kefir with high nutrient concentrations, which favored the growth of *L. nagelii* and *S. cerevisiae*. The presence of oxygen allowed the proliferation of AAB, resulting in high concentrations of acetic acid (Laureys et al., 2018). In addition, three main metabolic products were evaluated from the carbon flux from sucrose during 192 h of fermentation (Martínez-Torres et al., 2017). After 24 h, lactic and acetic acid have been postulated to be initially produced by *L. hilgardii* and subsequently produced by *Acetobacter* spp., mainly *A. tropicalis*. Ethanol was almost entirely oxidized to acetic acid, which could be further dissimilated by *Acetobacter* species.

However, these studies only determined total metabolite concentrations produced by the microorganisms during fermentation, but they did not reveal, how LAB, AAB and yeasts benefit from or affect each other through dynamic metabolite exchanges. Recently, we have shown that *L. hordei* TMW 1.1822 is highly adapted to the water kefir environment (Xu et al., 2019a) and its sucrose rich but amino- and fatty acids poor conditions. In the presence of abundant sucrose, it produces a dextran, which specifically induces the aggregation of *S. cerevisiae* as to ensure spatial proximity of the yeast cells in an initial step of granule formation (Xu et al., 2018). In a quantitative proteomic analysis we could quantify 233 differentially expressed proteins of *L. hordei* as its response to the co-culture with *S. cerevisiae* (Xu et al., 2019b). These were predicted to be involved in citrate and amino acids metabolism as well as maintenance of NAD^+^/NADH homeostasis. It appears that *L. hordei* benefits from *S. cerevisiae* by enhanced availability of amino acids, while it alleviates acid stress of the yeast via metabolism of arginine provided by the yeast.

In order to probe whether the response of *L. hordei* to *S. cerevisiae* and its role for the water kefir system are typical or unique as compared to other water kefir lactobacilli, we investigated *L. nagelii* and compared its response to co-culture with *S. cerevisiae* with that one of *L. hordei*.

## Materials and Methods

### Strain Culture, Whole-Genome Sequencing, and Cell Counts

*L. nagelii* TMW 1.1827 isolated from water kefir by Gulitz et al. (2011) was single-cultured anaerobically at 30°C in modified MRS (mMRS) medium (Stolz et al., 1995). Genomic DNA was isolated, as described previously (Xu et al., 2019a), and sent to GATC Biotech (Konstanz, Germany) for PacBio SingleMolecule RealTime sequencing. The whole genome sequences were annotated by the NCBI Prokaryotic Genome Annotation Pipeline and RAST, which is a SEED-based prokaryotic genome annotation service using default settings (Aziz et al., 2008; Overbeek et al., 2013), as described previously (Xu et al., 2019a), and their key features were summarized in Supplementary Table S1.

*S. cerevisiae* TMW 3.221 was pre-cultured in YPG medium (Xu et al., 2019b). Single-cultivated *S. cerevisiae, L. nagelii* and co-cultivated *L. nagelii* TMW 1.1827 and *S. cerevisiae* TMW 3.221 were prepared in water kefir medium (WKM) (Stadie et al., 2013). Cell counts were assessed by plating serial dilutions of co-cultivated *L. nagelii* and *S. cerevisiae* on mMRS agar plates, supplemented with cycloheximide and YPG agar plates, supplemented with chloromycetin, respectively. In the same way, single-cultivated *L. nagelii* was plated on mMRS agar plates and single-cultivated *S. cerevisiae* on YPG agar plates, as described previously by Xu et al. (2019b).

### Chromatographic Analysis of Amino Acids, Sugars, and Organic Acids

1% pre-cultured *L. nagelii* TMW 1.1827 and *S. cerevisiae* TMW 3.221 were separately inoculated into chemically defined medium (CDM) in triplicate, as described previously (Xu et al., 2019a). After 24 h of cultivation at 30°C, 1 ml of each culture and 1 ml of CDM as a control were mixed with 50 μl of 70% (v/v) perchloric acid (Sigma-Aldrich, St. Louis, MO, United States) and subsequently incubated overnight at 4°C for protein precipitation. After centrifugation (12,000 rpm, 10 min), the supernatant was collected and filtered by 0.2 μm Phenex^TM^ Regenerated Cellulose Membrane (Phenomenex, Aschaffenburg, Germany) for the detection of amino acids and organic acids as below. Amino acids were analyzed on a Dionex Ultimate 3000 HPLC system (Dionex, Idstein, Germany) using a Gemini C18 column (Phenomenex, Aschaffenburg, Germany) with UV detection at 338 and 269 nm. Quantification was executed employing calibration adjustment by external HPLC grade standards and the Chromeleon software version 6.80 (Dionex, Idstein, Germany).

Consumption and production of sugars and organic acids of *L. nagelii* and *S. cerevisiae* grown in CDM for 24 h were quantified by a Dionex UltiMate 3000 HPLC system (Dionex, Idstein, Germany) with Rezex ROA-Organic Acid H^+^ column (Phenomenex, Aschaffenburg, Germany) and RI-101 detector (Shodex, München, Germany), as described previously (Xu et al., 2019a). For sugar analysis, 500 μl of each sample were mixed with 250 μl of a 10% (w/v) ZnSO_4_^∗^7H_2_O solution and afterward added with 250 μl 0.5 M NaOH. After incubation for 20 min at 25°C, the supernatant was obtained by centrifugation and filtered as described above. Analytes were separated at a constant flow rate of 0.7 ml/min with a column temperature of 85°C for 30 min. Sulfuric acid (Rotipuran, Roth, Karlsruhe, Germany) solution with a concentration of 5 mM served as mobile phase.

### Proteomic Sample Preparation and Label-Free Quantitative Proteomic Analysis

Co-cultivated *L. nagelii* and *S. cerevisiae*, as well as single-cultured *L. nagelii* and *S. cerevisiae* were incubated anaerobically in WKM at 30°C for 10 h in triplicate and prepared for proteomic analysis, as previously described (Xu et al., 2019a). First of all, these samples were treated with trichloroacetic acid (TCA, 6.25% w/v), centrifuged (5,000 rpm, 5 min) at 4°C, washed with acetone and reconstituted in lysis buffer [8 M urea, 5 mM EDTA di-sodium salt, 100 mM (NH)_4_HCO_3_, 1 mM dithiothreitol (DDT)]. Subsequently, the cells were mechanically disrupted with acid-washed glass beads (G8772, 425–600 μm, Sigma, Germany). Proteins were reduced with 10 mM DTT at 30°C for 30 min, and subsequently carbamidomethylated with 55 mM chloroacetamide in the dark for 60 min. Finally, proteins were digested by trypsin and desalted by C18 solid phase extraction using Sep-Pak columns (Waters, WAT054960). Purified peptide samples were dried in a SpeedVac concentrator (Acid-Resistant CentriVap Vacuum Concentrator, Labconco) and resuspended in an aqueous solution containing 1.9% acetonitrile and 0.1% formic acid to a final concentration of 0.25 μg/μl.

Generated peptides were analyzed on a Dionex Ultimate 3000 nano LC system, coupled to a Q-Exactive HF mass spectrometer (Thermo Scientific, Bremen, Germany), as described previously (Xu et al., 2019b). Peptides were delivered to a trap column (75 μm × 2 cm, self-packed with Reprosil-Pur C18 ODS-3 5 μm resin, Dr. Maisch, Ammerbuch, Germany) at a flow rate of 5 μl/min in solvent A_0_ (0.1% formic acid in water). Peptides were separated on an analytical column (75 μm × 40 cm, self-packed with Reprosil-Gold C18, 3 μm resin, Dr. Maisch, Ammerbuch, Germany), using a 120 min linear gradient from 4 to 32% solvent B (0.1% formic acid, 5% DMSO in acetonitrile) and solvent A_1_ (0.1% formic acid, 5% DMSO in water) at a flow rate of 300 nl/min. The mass spectrometer was operated in data dependent mode, automatically switching between MS1 and MS2 spectra. MS1 spectra were acquired over a mass-to-charge (m/z) range of 360–1,300 m/z at a resolution of 60,000 (at m/z 200) using a maximum injection time of 50 ms and an AGC target value of 3e6. Up to 20 peptide precursors were isolated (isolation window 1.7 m/z, maximum injection time 25 ms, AGC value 1e5), fragmented by higher-energy collisional dissociation (HCD), using 25% normalized collision energy (Letort et al., 2002) and analyzed at a resolution of 15,000 with a scan range from 200 to 2,000 m/z.

To enable differentiation of *L. nagelii* and *S. cerevisiae* proteins and their identification, peptide and protein identification plus quantification were performed with MaxQuant (version 1.5.7.4) by searching the MS2 data against all protein sequences obtained from UniProt – reference proteome *S. cerevisiae* S288c (6,724 entries, downloaded 13.03.2017) and all protein sequences from *L. nagelii* TMW 1.1827 (cf. section “Comparative Genomic Features and Growth Characteristics of *L. nagelii* in the Presence of *S. cerevisiae*,” GenBank CP018180 – CP018183), using the embedded search engine Andromeda (Cox et al., 2011), as previously described (Xu et al., 2019a). Carbamidomethylated cysteine was a fixed modification. Oxidation of methionine, and N-terminal protein acetylation were variable modifications. Precursor and fragment ion tolerances were 10 ppm and 20 ppm, respectively. Label-free quantification and data matching between consecutive analyses were enabled within MaxQuant. Search results were filtered for a minimum peptide length of seven amino acids, 1% peptide and protein false discovery rate (FDR) plus common contaminants and reverse identifications. MaxQuant output files were further analyzed using Perseus (version 1.5.6.0) (Tyanova et al., 2016). iBAQ intensities were log_2_-transformed for further statistical analysis. NCBI annotation, PSORTb subcellular localization, SEED category (subcategory and subsystem) as previously annotated (cf. section “Strain Culture, Whole-Genome Sequencing, and Cell Counts”) were added to the matrix through identifier matching. For the comparison between two groups, *t*-tests were performed. Log_2_ fold change ≥ 2 or ≤-2 and -Log_10_ *P*-value ≥ 2 (*p-*value ≤ 0.05) were considered to be significantly differentially expressed proteins of *L. nagelii* TMW 1.1827 in the presence of *S. cerevisiae* TMW 3.221.

### Statistical Analysis and Visualization

A genomic atlas of *L. nagelii* TMW 1.1827 was generated using Artemis and DNA plotter^1^ (Carver et al., 2008) as described previously (Xu et al., 2019a). Subcellular localization of proteins was predicted, using the tool PSORTb (Version 3.0.2^2^) (Gardy et al., 2004; Yu et al., 2010). All the annotated EC numbers from RAST were imported into iPath 3.0^3^ (Yamada et al., 2011) for generating an overview of complete metabolic pathways and biosynthesis of other secondary metabolites.

The sucrose metabolism, pyruvate metabolism, and amino acid biosynthesis pathways of *L. nagelii* TMW 1.1827 were constructed based on the self-constructed overview on the key reactions involved in sucrose metabolism, pyruvate metabolism, and amino acid biosynthesis pathways of *L. hordei* TMW 1.1822 as described previously (Xu et al., 2019a). Enzymes involved in each reaction step were manually checked, whether they were present in translated open reading frames (ORFs) annotated from both, NCBI and RAST. The figure of the biosynthesis pathways of amino acids and riboflavin was generated using the KEGG PATHWAY mapping tool^4^ by importing EC numbers only involved in amino acid biosynthesis and riboflavin metabolism.

Genomic differences between *L. nagelii* TMW 1.1827 and *L. hordei* TMW 1.1822 were identified using Blast Diagnostic Gene findEr (BADGE) (Behr et al., 2016) under modified settings. The “min_DMG_occurance” was set to 0.00000000000001. The “megablast_perc_identity_cut” value was set to 90, while both, the “megablast_within_groub_qscov” and the “megablast_between_group_qscov” value was set to 0.90. The dc_mode was enabled. Additionally, BADGE was run on protein level using default protein-level options. The BADGE output was divided in pan and core genome. The genome comparison was graphically visualized by the BLAST Ring Image Generator (BRIG) (Alikhan et al., 2011) using the annotated and translated ORFs of the pan genome as reference. Furthermore, the genomic differences between *L. nagelii* TMW 1.1827 and *L. nagelii* DSM 13675 were identified by BADGE using default settings.

### Data Deposition

The whole-genome sequence of *L. nagelii* TMW 1.1827 was submitted to GenBank designated as BioSample SAMN06052354, referred to as accession numbers CP018180 to CP018183. An additional file containing all metadata of *L. nagelii* TMW 1.1827 from NCBI and RAST annotation is deposited as Supplementary Material. The mass spectrometry proteomics data have been deposited to the ProteomeXchange via the PRIDE partner repository with the dataset identifier PXD012513^5^.

## Results and Discussion

### Comparative Genomic Features and Growth Characteristics of *L. nagelii* in the Presence of *S. cerevisiae*

The genomic size of *L. nagelii* TMW 1.1827 is 2.41 Mbp and exhibits a GC content of 36.68% (shown in Table 1). *L. nagelii* TMW 1.1827 exhibits a total number of 2,391 coding sequences (CDS), including all three plasmids (shown in comparison with *L. hordei* in Table 1 and visualized in Supplementary Figure S1). So far, the only published whole genome sequences of *L. nagelii* strains result from a comparative genomics project together with 211 other LAB strains (Sun et al., 2015). *L. nagelii* DSM 13675 isolated from wine, was associated to a different environment than water kefir and therefore faces different conditions. Those differences in the adaptation to distinct environmental conditions were also displayed in the genomes. For the two *L. nagelii* strains from wine and water kefir the annotated differences could be referred to genes related to carbohydrate metabolism, namely enzymes of citrate and concomitant acetolactate metabolism, which were only found in the water kefir isolate *L. nagelii* TMW 1.1827. Also, the water kefir isolate differed from the wine isolate by galactose PTS and metabolism including the tagatose pathway. As citrate and galactose are present or absent, respectively, in both environments, a specific adaptation to the respective source of isolation cannot be deduced from this. The genomic reflection of environmental adaptation observed in strains of *L. hordei* isolated from widely different environments of malted barley (DSM 19519; Sun et al., 2015) or water kefir TMW 1.1822; Xu et al., 2019a), respectively, was more decisive and markedly resides in sucrose metabolism.

**Table 1:** Comparative genomic features of *L. nagelii* TMW 1.1827 with *L. hordei* TMW 1.1822.

	Genome length (Mbp)	GC content	Number of features	Total number of coding sequences plus plasmids	Total feature length (Mbp)	Coding density (%)
*L. nagelii* TMW 1.1827	2.41	36.68	2232	2461	2.10	87.18
*L. hordei* TMW 1.1822	2.42	35	2268	2391	2.09	86.27


**FIGURE 1:**
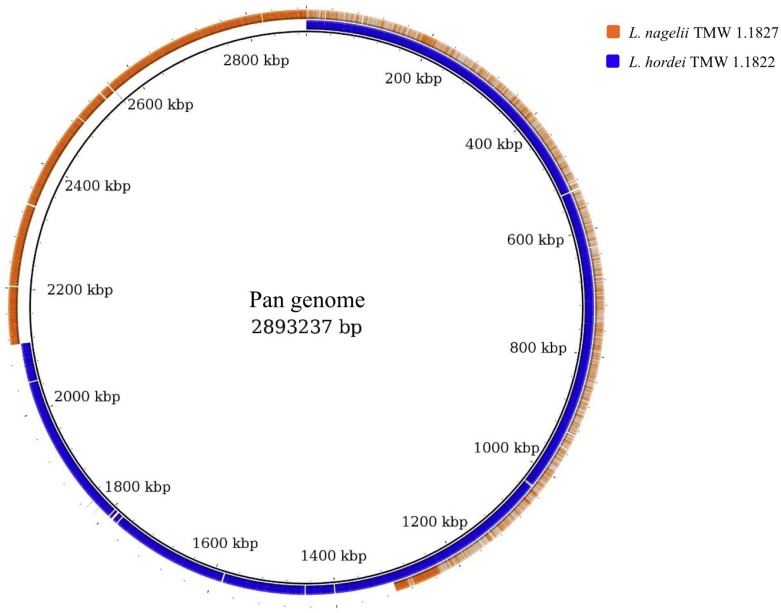
Whole genome comparison as visualized by BRIG (Alikhan et al., 2011). CDS of the pan genome was used as reference and the genomes of both microorganisms were aligned to this reference. As a result, the structures of the genomes and the pan genome did not reflect the physical structure of the chromosomes or plasmids. The core genome was approximately half of the pan genome and was detected from the beginning until about 1,300 kbp. Strain specific genes were displayed in the range of approximately 1,300 kbp until the end.

**FIGURE 2:**
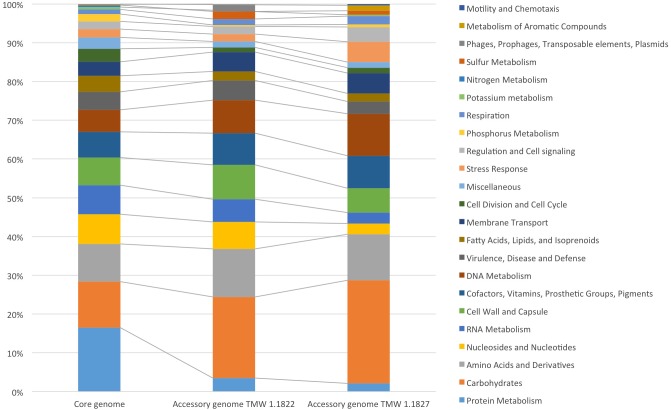
Annotated SEED categories of proteins, divided in the core and accessory genomes of *L. hordei* TMW 1.1822 and *L. nagelii* TMW 1.1827, which was done by BADGE analysis. The proportion of proteins assigned to each SEED category with respect to the total number of proteins is shown in the bar chart.

For comparative insights the whole genome sequences of *L. hordei* TMW 1.1822 and *L. nagelii* TMW 1.1827 were compared to each other using BADGE. As visualized in Figure 1, the core genome of both microorganisms included 1,380 CDS, which displays 56.0% of the whole genome of *L. hordei* TMW 1.1822 and 57.7% of the whole genome of *L. nagelii* TMW 1.1827. The main components of the core genome were found in the SEED categories of protein, carbohydrate and amino acid metabolism. The accessory genome of *L. hordei* TMW 1.1822 as compared to that one of *L. nagelii* TMW 1.1827 was dominated by additional genes for carbohydrate and amino acid metabolism, and cell wall biosynthesis. Corresponding results were found for *L. nagelii* TMW 1.1827, except for the SEED category of cell wall formation, which was substituted by CDS involved in DNA metabolism (shown in Figure 2). Since both microorganisms are associated to water kefir, representing an environment rich in sugar, it was not surprising, that *L. nagelii* TMW 1.1827 and *L. hordei* TMW 1.1822 mainly adapted to it by additional genes coding for carbohydrate metabolism.

**FIGURE 3:**
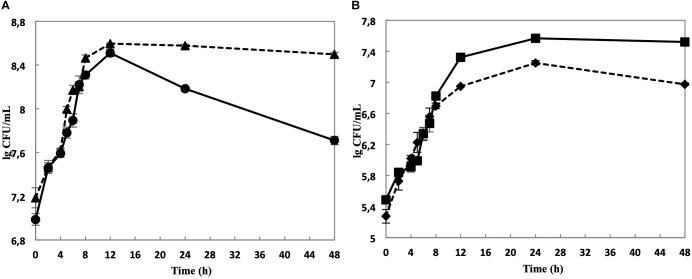
Cell counts of *L. nagelii* TMW 1.1827 in single culture (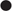) and co-cultivation with *S. cerevisiae* TMW 3.221 (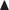) **(A)**. Cell counts of *S. cerevisiae* TMW 3.221 in single culture (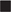) and in co-cultivation with *L. nagelii* TMW 1.1827 (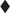) **(B)**.

While the cell yield of single cultivated *L. nagelii* TMW 1.1827 was only slightly increased upon co-cultivation with *S. cerevisiae* after 8 and 12 h of fermentation (Figure 3A), it declined significantly slower in co-cultivated *L. nagelii* as compared to single-cultivated *L. nagelii* until 24 h. I appears that the co-culture with *S. cerevisiae* preconditions *L. nagelii* toward an increased tolerance to the (e.g., increasingly acidic) environmental conditions. On the other hand, the cfu of *S. cerevisiae* were reduced upon co-cultivation with *L. nagelii* (Figure 3B). This indicates that *L. nagelii* TMW 1.1827 affects the growth of *S. cerevisiae* much more than *L. hordei* TMW 1.1822 (Xu et al., 2019b). To get insights into the reasons of these differences, prediction of dynamic metabolite exchanges were explored by proteomics in this study for *L. nagelii* and compared with those previously determined for *L. hordei* (Xu et al., 2019b).

### General Proteomic Analysis and Overview of Predicted Complete Metabolic Activities

**FIGURE 4:**
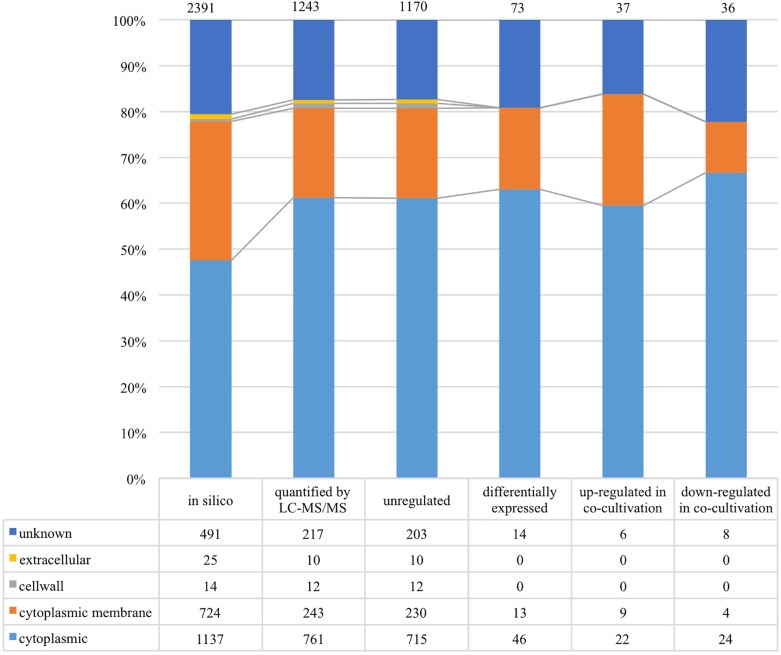
Subcellular localization of *L. nagelii* proteins (*in silico*, quantified by LC-MS/MS, unregulated, differentially expressed, up-regulated in co-cultivation, down-regulated in co-cultivation), which were predicted by PSORTb. The proportion of *L. nagelii* proteins assigned to each respective subcellular compartment and the group “unknown” with respect to the total number of *L. nagelii* proteins is shown by the bar chart. The table below shows the respective absolute numbers.

**FIGURE 5:**
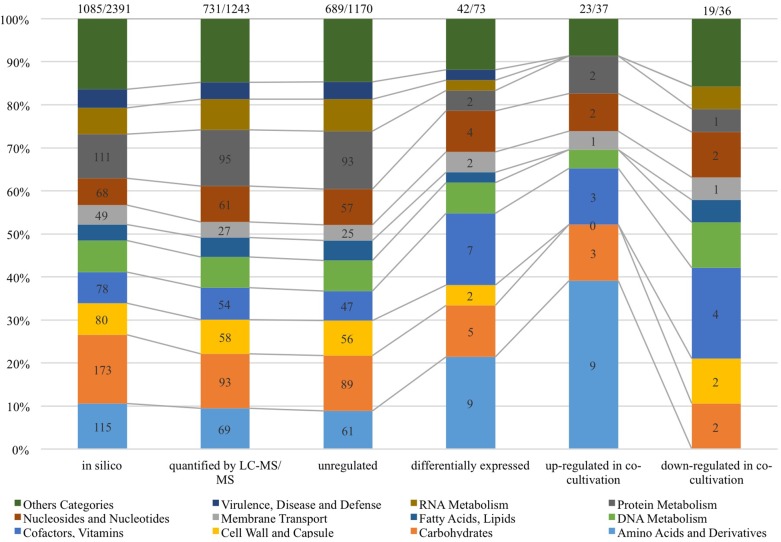
SEED categories of *L. nagelii* proteins (*in silico*, quantified by LC-MS/MS, unregulated, differentially expressed, up-regulated in co-cultivation, down-regulated in co-cultivation), which were predicted by SEED. The proportion of *L. nagelii* proteins assigned to each respective category of metabolism and the group “other categories” which is the sum of several small categories with respect to the total number of *L. nagelii* proteins is shown by the bar chart. The ratio on the top of each column is the number of predicted SEED categories accounts for the number of all coding DNA sequence (CDS).

As shown in Figure 4, 1,243 proteins of *L. nagelii* TMW 1.1827 were identified and quantified by proteomic analysis, comprising about 52% of the genes annotated by whole genome analysis. A comprehensive overview of the complete metabolic pathways and significantly differentially expressed (DE) proteins of *L. nagelii* in the presence of *S. cerevisiae* is provided in Supplementary Figure S2. As shown in Figure 5, there were 73 DE proteins in *L. nagelii* regulated in the presence of *S. cerevisiae*. Those up/down-regulated proteins of *L. nagelii* were most abundant in the SEED categories “amino acids and derivatives” (9 out of 69), “carbohydrates” (5 out of 93) “nucleosides and nucleotides” (4 out of 61) and “cofactors, vitamins” (7 out of 54) (shown in Figure 5).

### Sugar Transport and Carbohydrate Metabolism

**FIGURE 6:**
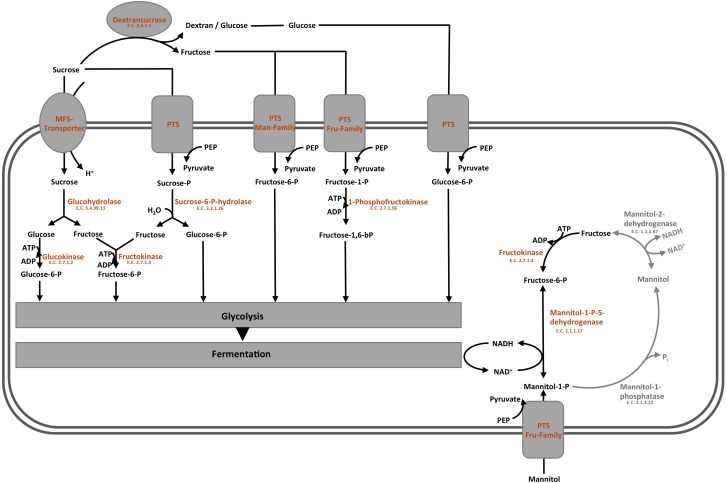
Modified overview on the key reactions involved in sucrose metabolism (Xu et al., 2019a) of *L. nagelii* TMW 1.1827 in the presence of *S. cerevisiae* TMW 3.221: the enzyme colored in blue was annotated by genomics but not quantified by proteomics, the enzymes colored in orange were both annotated by genomics and quantified by proteomics, the enzymes colored in gray were neither annotated by genomics nor quantified by proteomics.

The overview on the key reactions involved in sucrose metabolism of *L. nagelii* is provided in Figure 6. *L. nagelii* encoded and expressed an MFS-transporter specific for sucrose uptake. As previously demonstrated, *L. nagelii* also produces a glucan from sucrose by an extracellular glucansucrase (Xu et al., 2018). The residual fructose can then be transported into the cell by a fructose specific PTS and simultaneous phosphorylation. Once inside the cell, the phosphorylated fructose can directly enter the glycolytic pathway. All PTS, namely for sucrose, glucose, fructose, mannose, sorbose, and mannitol uptake, were identified by proteomic analysis as constitutively expressed upon co-culture. Whole genome sequence analysis of *L. nagelii* TMW 1.1827 confirmed the presence of the genes encoding all enzymes required for the EMP and PKP pathways (locus tags and IDs given in Supplementary Table S2). Thus, *L. nagelii* TMW 1.1827 should also be considered as facultatively heterofermentative, such as *L. plantarum* WCFS1 and *Lactococcus lactis* (Kleerebezem and Hugenholtz, 2003; Kleerebezem et al., 2003). This is contrary to the fact that *L. hordei* DSM 19519 and *L. nagelii* DSM 13675 were inferred as obligately homofermentative strains according to their phenotype (Sun et al., 2015). However, those strains have been isolated from different environments.

To probe the principal fermentation type of *L. nagelii* TMW 1.1827 we determined fermentation metabolites upon its growth in CDM to find 40.1 mM lactate and 6.9 mM acetate after 24 h of fermentation. This way we could show for *L. nagelii* and also (previously) for *L. hordei* (Xu et al., 2019a) that the water kefir isolates of these species are indeed different from other ones even with respect to basic fermentation types. The data corroborate a homofermentative metabolism, in which energy generation via EMP and recycling of NAD^+^ by reducing pyruvate to lactate is favored. The small amount of acetate may reside from pyruvate by either generating formate via pyruvate formate lyase or by NADH and CO_2_ generation via the pyruvate dehydrogenase complex. Subsequently, the resulting acetyl-CoA may be metabolized to acetate. However, the latter option requires subsequent NAD^+^ recycling.

**FIGURE 7:**
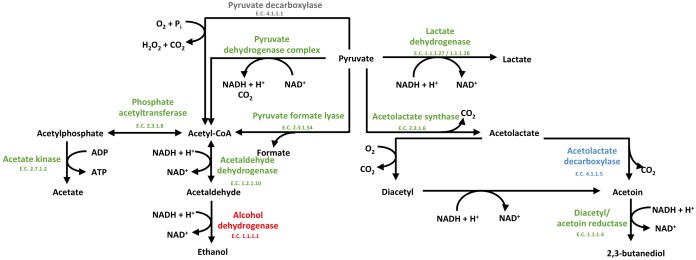
Modified predicted outline of pyruvate metabolism (Xu et al., 2019a) of *L. nagelii* TMW 1.1827 in the presence of *S. cerevisiae* TMW 3.221. The enzymes colored in red represented the up-regulated ones, while blue represented down-regulated proteins. Un-regulated proteins are indicated in green and proteins, neither annotated in the genome or proteome are colored in gray.

In the presence of *S. cerevisiae*, the 3-phosphoglycerate mutase of *L. nagelii* TMW 1.1827 was significantly down-regulated. As postulated previously, the expression of this enzyme is linked to the concentration of its substrate 3-phosphoglycerate (Smeianov et al., 2007). This indicates, that intermediates of early glycolytic steps may be used for other metabolic reactions or hexoses may rather enter PKP or PPP than EMP, resulting in less production of 3-phosphoglycerate. At the same time, the alcohol dehydrogenase (EC 1.1.1.1, 3.5 log_2_ fold change) of *L. nagelii* was significantly up-regulated in the presence of *S. cerevisiae* (shown in Figure 7). Yielding less ATP, but more reductive power, this metabolic switch to ethanol production may be important to keep the PKP running. *L. nagelii* TMW 1.1827 is capable of transporting and phosphorylating mannitol, possibly delivered by *S. cerevisiae*, inside the cell by a specific PTS and subsequent oxidation to fructose-6-P via mannitol-1-P-5-dehydrogenase. This provides evidence for the enhanced ethanol production as a recycling mechanism for the NADH, which is generated upon mannitol oxidation. Since fructose-6-P must not be phosphorylated prior to entering the EMP or PKP, there is less need for ATP generation upon acetate formation.

The expression of glucose-6-phosphate dehydrogenase, which is part of the PKP and PPP, was significantly up-regulated (Xu et al., 2019b). As discussed previously, this metabolic switch from EMP to PKP/PPP may also help *L. nagelii* to utilize gluconate, which appears to be a decisive trait in the water kefir environment (Xu et al., 2019a). Looking at the metabolic phenotype of other abundant LAB species in water kefir, these results are in line with the findings of Laureys et al. *L. paracasei*, which is the most dominant one during their water kefir grain growth (Laureys et al., 2018), is also facultatively heterofermentative lactobacilli as *L. hordei* TMW 1.1822 and *L. nagelii* TMW 1.1827.

Furthermore, α-acetolactate decarboxylase (-2.2 log_2_ fold change, shown in Figure 7) was significantly down-regulated in *L. nagelii*, blocking the direct decarboxylation of acetolactate to acetoin. Under aerobic conditions, acetolactate spontaneously decomposes into diacetyl enabling regeneration of two molecules of NAD^+^ upon reduction to 2,3-butanediol via diacetyl/acetoin reductase. Since oxygen is probably limited for *L. nagelii* due to the subsidence of the water kefir granules, this pathway for NAD^+^ regeneration may be completely disabled in the water kefir environment.

In contrast to *L. nagelii, L. hordei* possesses PTS specific for β-glucoside and cellobiose transport, and the expression of PTS belonging to the mannose–fructose–sorbose family were significantly up-regulated in *L. hordei.* This indicates a more restricted use of sugars by *L. nagelii.* The expression of EMP specific enzymes of *L. hordei* TMW 1.1822 was not influenced by *S. cerevisiae.* More decisively, *L. hordei* reacts to the presence of *S. cerevisiae* by down-regulation of alcohol dehydrogenase and up-regulation of diacetyl/ acetoin reductase and α-acetolactate decarboxylase, yielding 2,3-butanediol and NAD^+^ (Xu et al., 2019b). In conclusion, *L. nagelii* TMW 1.1827 displayed an antidromic strategy to maintain NAD^+^/NADH homeostasis after the metabolic switch induced by *S. cerevisiae* as compared to *L. hordei* TMW 1.1822.

Both, genomic and proteomic analyses revealed an incomplete TCA cycle in *L. nagelii.* In the case of *L. nagelii*, aconitate hydratase (EC 4.2.1.3), which catalyzes the stereo-specific isomerization of citrate to isocitrate via *cis*-aconitate, and isocitrate dehydrogenase (EC 1.1.1.42), which catalyzes the oxidative decarboxylation of isocitrate, producing 2-oxoglutarate and CO_2_, were significantly up-regulated (4.5 log_2_ fold change, 2.0 log_2_ fold change). Despite its incompleteness, the TCA cycle is an important supplier for compounds involved in other metabolic reactions and thus, isocitrate and 2-oxoglutarate may be useful for amino acid metabolism in *L. nagelii.*

Water kefir is a challenging environment for its habitants regarding low nutrient concentrations except for the excess sugar. Since lemon slices are added, it is not surprising that microorganisms in water kefir use citrate as a nutrient. *L. nagelii* is capable of direct citrate import using malate permease. Once inside the cell, citrate is converted by citrate lyase segregating one molecule of acetate. The resulting oxaloacetate may then be decarboxylated via oxaloacetate decarboxylase yielding pyruvate or is further used for amino acid biosynthesis. In contrast to *L. nagelii*, these enzymes are DE in *L. hordei*, which appears to be positively influenced in its metabolism of citrate as an additional carbon source upon co-culture with *S. cerevisiae* (Xu et al., 2019b). This may help to explain, why *L. hordei* is more abundant in the water kefir consortium than *L. nagelii* (Gulitz et al., 2011).

### Amino Acids Biosynthesis, Metabolism, and Transport

The concentration of amino acids in pure WKM is very low (<0.004 mmol/l, respectively) (Stadie et al., 2013). So respective metabolite quantification is way too low to obtain conclusive data on amino acids metabolism in WKM, namely on those metabolites, which are determinative for interaction of lactobacilli and yeasts. Indeed, this is a major reason to use quantitative proteomics for metabolic predictions. The *in silico* analysis of the genome and proteome of *L. nagelii* TMW 1.1827 did not reveal any known homologs of a cell wall proteinase (Prt). *L. nagelii* encodes the complete oligopeptide transport system OppABCDF (Tynkkynen et al., 1993; Detmers et al., 1998). Except from OppB, all genes of both annotated OppABCDF clusters were found to be present in the proteome of *L. nagelii.* Despite lacking an expressed OppB, the growth of *L. nagelii* was not impaired. This phenomenon was already described for other bacteria (Nepomuceno et al., 2007) indicating, that the function of OppB may be compensable by other *trans*-membrane proteins. In the presence of the yeast, the remaining proteins were widely un-regulated with the exception of OppF, which was significantly down-regulated in one cluster. Since OppF is responsible for coupling the energy of ATP hydrolysis with the import of oligopeptides, *L. nagelii* may reduce energy consumption caused by oligopeptide uptake. In contrast, *L. hordei* upregulated its OppABCDF system and a set of peptidases, suggesting that *L. hordei* benefits from peptides, which are more readily available in the presence of the yeast (Xu et al., 2019b). Since water kefir provides very limited resources of proteins and free amino acids, mainly originating from dried fruits and the yeast, these findings may also explain the fact, that the growth of *L. hordei* is stimulated in co-culture.

**FIGURE 8:**
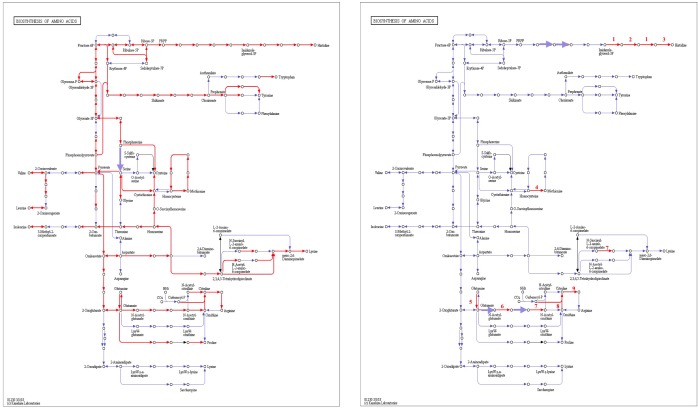
Biosynthesis of amino acids of *L. nagelii* TMW 1.1827 **(A)** and *L. nagelii* TMW 1.1827 in presence of *S. cerevisiae* TMW 3.221 **(B)**. In **(A)**, the red arrowed lines show the presence of enzymes annotated from genome. In **(B)**, the red arrowed lines indicate up-regulated proteins.

From genomic annotation, *L. nagelii* encodes several amino acid permeases and transporters. In the presence of *S. cerevisiae*, methionine aminopeptidase and amino acid permease were significantly up-regulated, suggesting that the yeast induces amino acid uptake in *L. nagelii.* However, it was not possible to specify from sequence comparison, which amino acids were ingested by *L. nagelii.* Still, this may be solved by a closer look at amino acid synthesis pathways and auxotrophies. The genomic analysis of *L. nagelii* TMW 1.1827 revealed the prototrophy for 13 amino acids and auxotrophy for 7 amino acids (Table 2). According to the quantitative proteomic analysis, nine enzymes of *L. nagelii* involved in histidine, methionine, glutamate, and arginine biosynthesis pathways were all significantly up-regulated in the presence of *S. cerevisiae* (shown in Figure 8 and Table 3). Since those biosynthesis pathways can also be used for amino acid catabolism, water kefir microorganisms may profit from amino acids secreted by the yeast, creating a symbiotic consortium. However, from *in silico* analysis, the direction of a respective metabolic pathway remains speculative. Still, together with physiological data on amino acid consumption and secretion of *L. nagelii* and *S. cerevisiae*, this can be solved for at least some of the predicted cases.

**Table 2:** List of the auxotrophies of *L. nagelii* TMW 1.1827.

Name of amino acid	Biosynthesis based on genome	Absent enzyme of biosynthesis of amino acid pathway	EC number
Alanine	P	None	
Arginine	P	None	
Asparagine	P	None	
Aspartic acid	P	None	
Cysteine	P	None	
Glutamic acid	P	None	
Glutamine	P	None	
Glycine	A	Phosphoserine phosphatase Threonine aldolase	EC 3.1.3.3EC 4.1.2.48
Serine	A	Phosphoserine phosphatase	EC 3.1.3.3
Histidine	P	None	
Leucine	A	Ketol-acid reductoisomerase Dihydroxy-acid dehydratase	EC 1.1.1.86EC 4.2.1.9
Isoleucine	A	Citramalate synthaseKetol-acid reductoisomerase Dihydroxy-acid dehydratase	EC 2.3.1.182 EC 1.1.1.86EC 4.2.1.9
Valine	A	Ketol-acid reductoisomerase Dihydroxy-acid dehydratase	EC 1.1.1.86EC 4.2.1.9
Lysine	P	None	
Methionine	P	None	
Proline	P	None	
Phenylalanine	A	Prephenate dehydratase Aromatic-amino-acid transaminase	EC 4.2.1.51EC 2.6.1.57
Tryptophan	A	Anthranilate phosphoribosyltransferase Anthranilate synthase Indole-3-glycerol phosphate synthase Phosphoribosylanthranilate isomerase	EC 2.4.2.18EC 4.1.3.27EC 4.1.1.48EC 5.3.1.24
Tyrosine	P	None	
Threonine	P	None	


*P represents prototrophy for amino acids, while A represents auxotrophy for amino acids.*

**Table 3:** Significantly differentially expressed proteins in *L. nagelii* TMW 1.1827 in response to *S. cerevisiae* TMW 3.221 involved in amino acid biosynthesis.

Number	Enzyme	EC number	Log_2_ fold change (co-cultivation vs. single culture)	-Log (*P*-value)	SEED subcategory
Up-regulated					
1	Imidazoleglycerol-phosphate dehydratase	EC 4.2.1.19	4.6	3.5	Histidine biosynthesis
2	Histidinol-phosphate aminotransferase	EC 2.6.1.9	4.4	3.8	
3	Histidinol dehydrogenase	EC 1.1.1.23	5.3	4.5	
4	5-Methyltetrahydropteroyltriglutamate -homocysteine methyltransferase	EC 2.1.1.14	3.8	3.7	Methionine biosynthesis
5	Glutamate synthase	EC 1.4.1.13	4.1	2.5	Glutamate, arginine biosynthesis
6	Acetylglutamate kinase	EC 2.7.2.8	3.0	1.7	Arginine biosynthesis
7	Acetylornithine aminotransferase	EC 2.6.1.11	8.7	3.3	
8	Ornithine carbamoyltransferase	EC 2.1.3.3	7.5	2.9	
9	Argininosuccinate synthase	EC 6.3.4.5	3.3	3.6	


**FIGURE 9:**
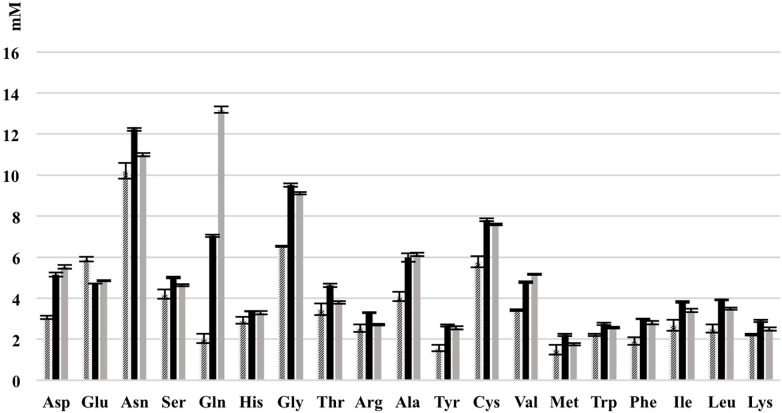
Consumption of amino acids of *L. nagelii* TMW 1.1827 and *S. cerevisiae* TMW 3.221 isolated from water kefir grown in CDM after 24 h. Black bar represents CDM, slash bar represents *L. nagelii*, gray bar represents *S. cerevisiae.*

As shown most prominently in Figure 9, *S. cerevisiae* secreted glutamine in high amounts, whereas *L. nagelii* consumed the amino acid at high levels via an up-regulated amino acid permease involved in glutamine uptake. This suggests, that *L. nagelii*, even though it is capable of producing glutamine by itself, profits from the glutamine provided by the yeast via the up-regulated glutamate synthase. Since glutamine plays an important role in anaplerotic sequences of transamination reactions in the biosynthesis of other amino acids, and also as a nitrogen carrier for the production of amino sugars and nucleotides, the uptake of this amino acid may be crucial to persist in the water kefir environment. *L. nagelii* was predicted to produce glutamate by itself via the up-regulated glutamate synthase using glutamine and 2-oxoglutarate, which probably results from the incomplete TCA cycle. This was consistent with an un-regulated glutamine synthetase in the presence of *S. cerevisiae.* As already described for *Lactobacillus crispatus* ST1, this enzyme might exhibit additional functions, if displayed on the bacterial surface, which enable physical coherence of the water kefir consortium under stressful conditions (Kainulainen et al., 2012). As a result, the yeast aggregation promotion of *L. hordei* by its functional dextran (Xu et al., 2018) may be even enhanced by over expression of this enzyme. Furthermore, among all amino acids, the production of glutamate is of primary importance in the assimilation of nitrogen, representing a donor for amino groups in the synthesis of other amino acids (Bernard and Habash, 2009; Dincturk et al., 2011).

Other amino acids were not produced, but partly consumed by the yeast after 24 h of fermentation in CDM. Therefore, it was not possible to determine real-time metabolic exchange (release/uptake) between *L. nagelii* and *S. cerevisiae* based on physiological data. Still, the label-free quantitative proteomic analysis enabled the investigation of the dynamic metabolic exchanges between microbial communities in water kefir. The DE enzymes involved in amino acid biosynthesis or catabolism predict that *S. cerevisiae* releases glutamine, histidine, methionine, and arginine, which are subsequently used by *L. nagelii* to ensure its survival in the water kefir consortium.

### Acid Tolerance by ADI Pathway

Functionally, the ADI pathway enables enhanced acid tolerance and energy provision in a variety of LAB genera such as *Lactobacillus, Lactococcus, Leuconostoc*, and *Weissella* (Tonon and Lonvaud-Funel, 2002; Fernández and Zúñiga, 2006; Rimaux et al., 2011). The system involves the three enzymes arginine deiminase (ADI), ornithine transcarbamylase (OTC), carbamate kinase (CK) and a transmembrane arginine/ornithine antiporter, which exchanges extracellular arginine against intracellular ornithine. While ADI and OTC were present in both, the genome and proteome of *L. nagelii* TMW 1.1827, CK and the arginine/ornithine antiporter were only detectable in the genome. Thus, it should be unable to convert carbamoyl-P to generate additional ATP in co-culture. In the energy rich environment of water kefir, this does not appear to be a disadvantage. Therefore, the fate of carbamoyl-P remains unclear. However, only OTC was significantly up-regulated in *L. nagelii* in co-culture with *S. cerevisiae.* This reaction may occur in both directions yielding citrulline or ornithine and carbamoyl-phosphate. *L. nagelii* did not encode any complete alternative acid tolerance systems, e.g., the agmatine deiminase (AGDI) system or the glutamate decarboxylase (GAD) system. Except for neutralization upon ammonia formation via the ADI system, acidification appears limited by the switch from lactic and acetic acid production to ethanol formation, when *L. nagelii* and *S. cerevisiae* were co-cultivated.

In contrast, all respective enzymes of *L. hordei* involved in ADI pathway were up-regulated in co-culture with the yeast. Although the fate of carbamoyl-phosphate and other incidental compounds remains unclear, *L. hordei* likely produces ammonia upon arginine hydrolysis to protect itself from pH stress by alkalization of its cytoplasm and proximal environment. Consequently, only *L. hordei* should reduce the acid stress for the yeast (Xu et al., 2019a).

### Fatty Acid Biosynthesis and Riboflavin Metabolism

Another limit in the water kefir environment is the limited availability of fatty acids. *L. nagelii* TMW 1.1827 appears to be deficient in FabB, which is a well studied 3-ketoacyl-ACP synthase for catalyzing the elongation reaction of fatty acid synthesis (Feng and Cronan, 2009), and additionally in FabA, which is hydroxyldecanoyl-ACP dehydratase/isomerase for the production of unsaturated fatty acids by many bacteria (Magnuson et al., 1993; Cronan and Rock, 1996). As demonstrated by Wang and Cronan (2004), FabF can functionally replace FabB, while FabZ adopts the function of FabA. It was also reported that expression of *Lactococcus lactis* FabF can functionally replace both FabB and FabF in *E. coli* (Morgan-Kiss and Cronan, 2008). Due to low sequence homologies, those enzymatic bi-functionalities are not predictable by genome analysis. Since both microorganisms grew to high cell densities in water kefir medium without any external fatty acids, those findings might also indicate the existence of other functional homologs for FabB and FabA in *L. nagelii.* Co-cultivation with *S. cerevisiae* does not alter the expression of any proteins involved in the fatty acid metabolism in both LAB (Supplementary Table S3). This indicates, that the beneficial effects of *S. cerevisiae* do not reside in a bilateral supply with unsaturated fatty acids. This situation resembles the one in *L. hordei*, which only lacks FabA but also should express functionally complementary alternatives (Xu et al., 2019a).

Moreover, there was a group of enzymes of *L. nagelii*, which showed decreased expression in response to the co-cultivation with *S. cerevisiae*, which are involved in the biosynthesis of riboflavin (as shown in Supplementary Figure S3). Riboflavin synthase (EC 2.5.1.9), 6,7-dimethyl-8-ribityllumazine synthase (EC 2.5.1.78), 5-amino-6-(5-phosphoribosylamine) uracil reductase (EC 1.1.1.193), GTP cyclohydrolase II (EC 3.5.4.25) and 3,4-dihydroxy-2-butanone 4-phosphate synthase (EC 4.1.99.12) were down-regulated in a range from -3.2 to -4.0 log_2_ fold. Those enzymes connect the purine metabolism and pentose phosphate pathway to synthesize riboflavin. Since generally yeast produce group B vitamins (Emery et al., 1946; Zeidler et al., 2002), and riboflavin production by some lactobacilli (such as *L. plantarum* and *L. fermentum*) was inducible (Burgess et al., 2006; Arena et al., 2014; Russo et al., 2014), this may be an evidence for the feeding of riboflavin from *S. cerevisiae* to *L. nagelii*, supporting its growth and leading to a stable water kefir consortium.

## Conclusion

The label-free quantitative approach represents a powerful tool for the identification and quantification of proteins to study the bacteria–yeast interaction of microorganisms involved in food fermentation processes (Behr et al., 2007; Siragusa et al., 2014; Maeda et al., 2015). It may even be used to explore more complex combinations or the complete water kefir system. However, with several (closely related) lactobacilli/yeasts in the system the sorting of proteins to species along sequence homologies will probably be limited because of sequence similarities across species. So in turn one would probably not be able to see the specific *L. nagelii/L. hordei* responses to *S. cerevisiae* any more, which are markedly different. So the reduction of the system offers also some advantage for a deeper understanding.

The predicted functional genome and the differentially expressed proteins in the presence of *S. cerevisiae* TMW 3.221 depicted the adaption of *L. nagelii* TMW 1.1827 to the water kefir consortium and environment, although protein regulations were less distinct than in *L. hordei* TMW 1.1822 (Xu et al., 2019b). Both microorganisms are highly efficient in degrading sucrose by an extracellular glucansucrase and subsequent fructose uptake, which may then enter EMP, PKP or mannitol metabolism. As already described for *L. hordei*, also *L. nagelii* appears to favor PKP over EMP, indicating a metabolic switch induced by an altered redox potential in the presence of *S. cerevisiae*. While *L. nagelii* remained widely un-affected in its citrate metabolism, the yeast stimulated *L. hordei* to use citrate as additional carbon source and therefore, promoting its growth.

Both LAB profit from glutamine secreted by the yeast, whereas *L. hordei* also takes advantage of the provided glutamate. While *L. hordei* up-regulated all of its enzymes involved in the reduction of acid stress via ADI pathway, *L. nagelii* only altered the expression of OTC. It was obvious, that both microorganisms reduced external acid stress by switching from lactate and acetate production to butanediol formation in the case of *L. hordei* and ethanol production in the case of *L. nagelii*.

At first glance, the fatty acid metabolism of both microorganisms appears to be impaired by the lack of one or more genes coding for key fatty acid biosynthesis enzymes. As it was already reported for other bacteria (Wang and Cronan, 2004), it is likely, that the functional role of those enzymes may be undertaken by other enzymes of the fatty acid biosynthesis gene cluster. This would explain, why both, *L. hordei* and *L. nagelii*, grew to high cell densities while facing an environment insufficient in unsaturated fatty acids. While *S. cerevisiae* TMW 3.221 modulated the protein expression of *L. hordei* TMW 1.1822 mainly in its carbohydrate metabolism, *L. nagelii* TMW 1.1827 seems to profit from secreted riboflavin. With respect to the establishment of a consortium maintaining physical proximity of lactobacilli and yeasts *L. hordei* appears to have a more prominent role as compared to *L. nagelii* as a result of its unique dextran causing yeast aggregation and proteins involved in adhesion functions.

## Data Availability

The datasets generated for this study can be found in GenBank, CP018180–CP018183, and the ProteomeXchange via the PRIDE partner repository with the dataset identifier PXD012513.

## Author Contributions

DX conducted the wet lab experiments and performed the primary data analysis. CL helped with proteomic data analyses and deposition. JlB conducted detailed analysis and metabolic predictions. JgB supervised data analyses. DX and JlB wrote the first draft of manuscript. JgB and RV established general layout of experimental approach, supervised DX and JlB and did final discussions and shaping of the manuscript.

## Conflict of Interest Statement

The authors declare that the research was conducted in the absence of any commercial or financial relationships that could be construed as a potential conflict of interest.

## Footnotes

1 http://www.sanger.ac.uk/science/tools/artemis

2 http://www.psort.org/psortb/

3 https://pathways.embl.de/ipath3.cgi?map=metabolic

4 http://www.genome.jp/kegg/tool/map_pathway1.html

5 http://proteomecentral.proteomexchange.org

## Supplementary Material

FIGURE S1 Genomic atlas of *L. nagelii* TMW 1.1827. Forward CDS (red), reverse CDS (blue), pseudogenes on both strands (black), tRNA and rRNA (dark green), % GC plot (yellow, high GC spike and green, low GC spike), GC skew [(G - C)/(G + C)] (gray).

FIGURE S2 Overview of enzymatic activity of *L. nagelii* TMW 1.1827 in the complete metabolic and other pathways in presence of *S. cerevisiae* TMW 3.221: the nodes colored in bold red represent up-regulated, in bold blue represent down-regulated enzymes or proteins according to proteomic data, while nodes colored in thin red represent all the rest enzymes or proteins according to genomic annotation data presented in iPath 3.0.

FIGURE S3 Overview of riboflavin metabolism of *L. nagelii* TMW 1.1827 generated in KEGG mapper. The EC numbers colored in red show up-regulated enzymes of *L. nagelii* in the presence of *S. cerevisiae*.

TABLE S1 Annotated key features of the *L. nagelii* TMW 1.1827 genome.

TABLE S2 List of annotated enzymes by NCBI and RAST involved in carbohydrate metabolism (glycolysis, pentose phosphate pathway, pyruvate metabolism, and TCA cycle).

TABLE S3 List of enzymes involved in fatty acid biosynthesis.
