# Supplementary figures and images for: Proteomic Analysis of Lactobacillus nagelii in the Presence of Saccharomyces cerevisiae Isolated From Water Kefir and Comparison With Lactobacillus hordei
Source: Front Microbiol. 2019 Feb 28;10:325. doi: 10.3389/fmicb.2019.00325 (PMC6413804; doi:10.3389/fmicb.2019.00325)

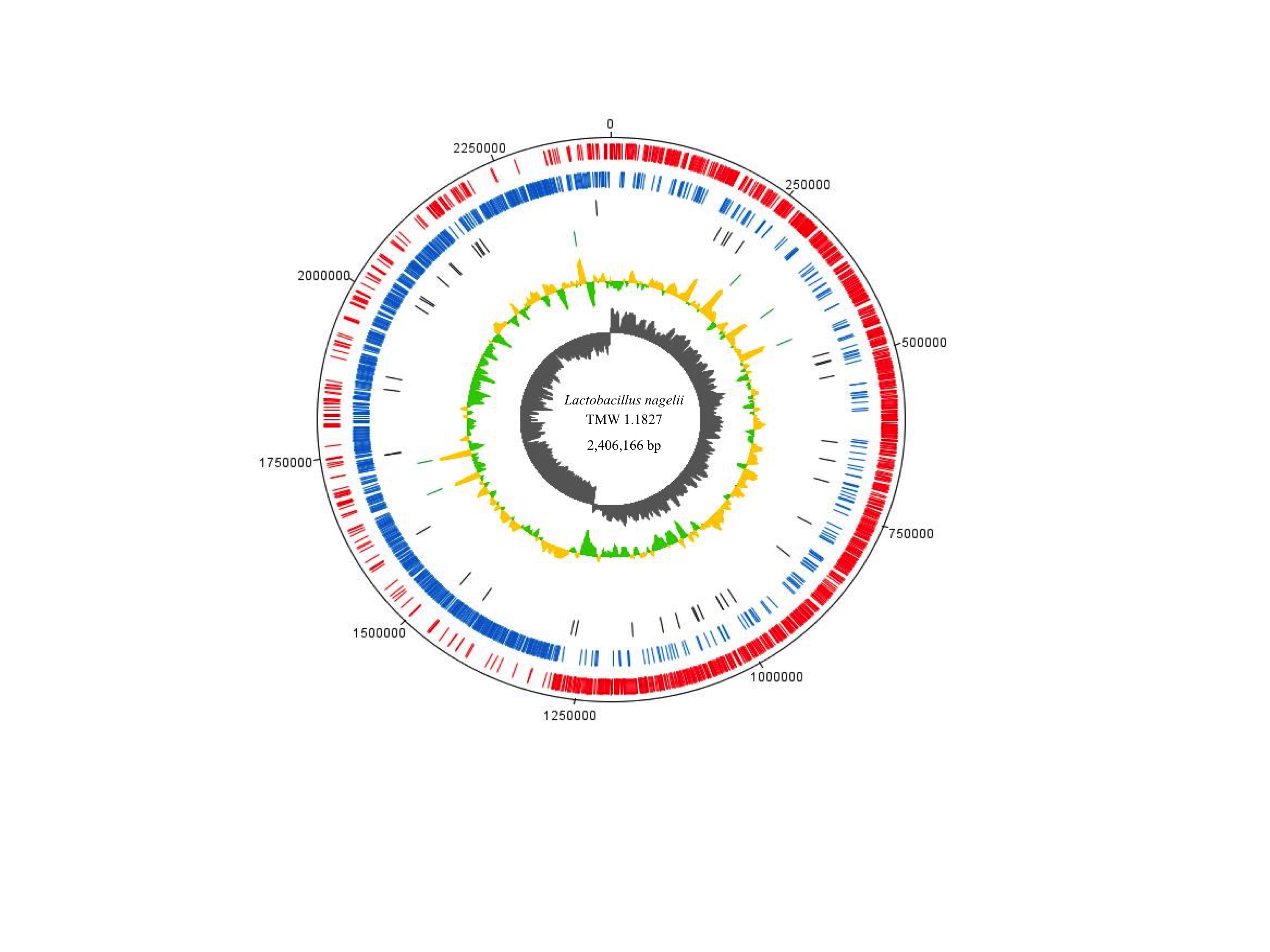

Supplement: FIGURE S1 — Genomic atlas of L. nagelii TMW 1.1827. Forward CDS (red), reverse CDS (blue), pseudogenes on both strands (black), tRNA and rRNA (dark green), % GC plot (yellow, high GC spike and green, low GC spike), GC skew [(G - C)/(G + C)] (gray). [file Image_1.JPEG]

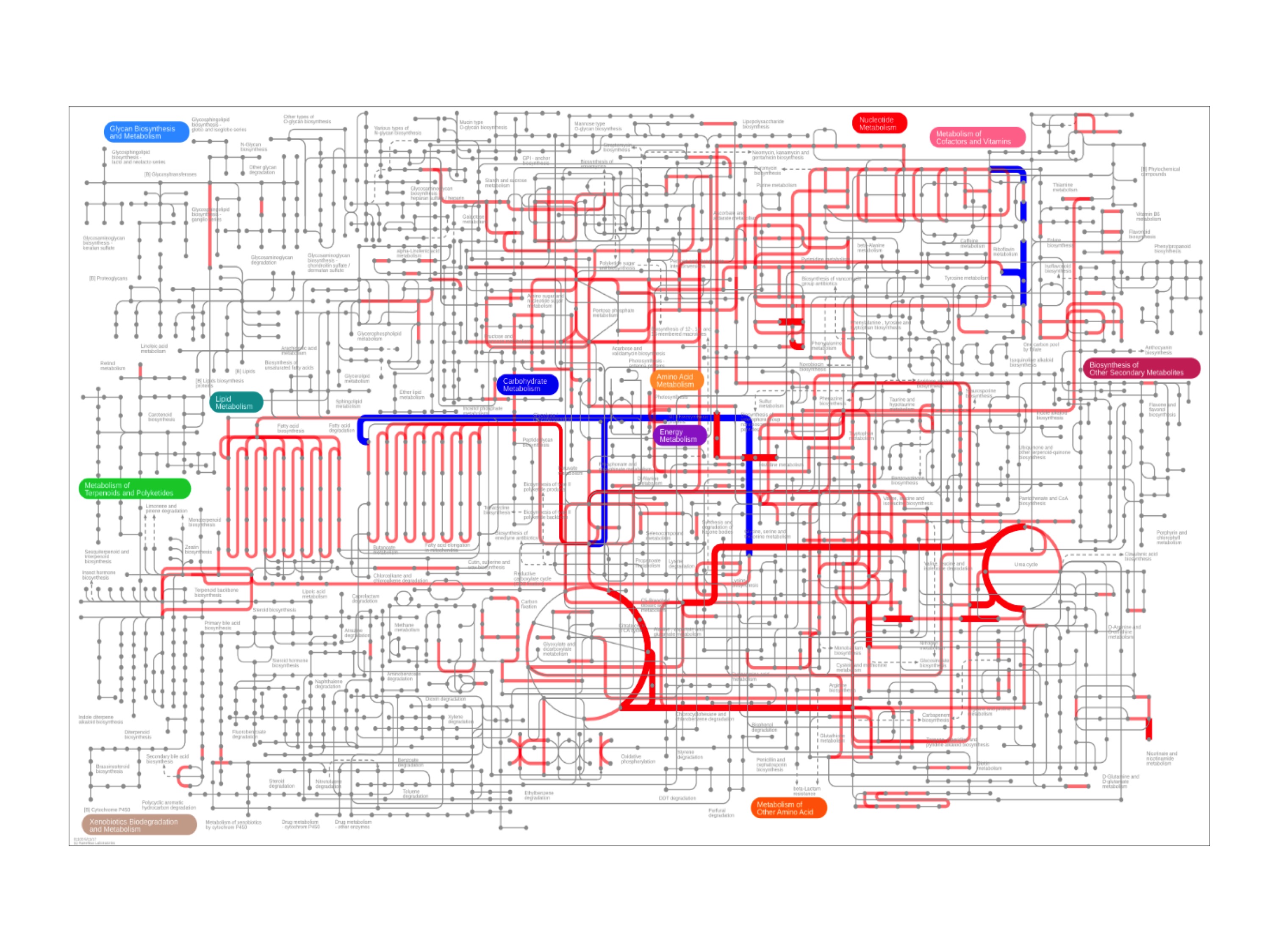

Supplement: FIGURE S2 — Overview of enzymatic activity of L. nagelii TMW 1.1827 in the complete metabolic and other pathways in presence of S. cerevisiae TMW 3.221: the nodes colored in bold red represent up-regulated, in bold blue represent down-regulated enzymes or proteins according to proteomic data, while nodes colored in thin red represent all the rest enzymes or proteins according to genomic annotation data presented in iPath 3.0. [file Image_2.JPEG]

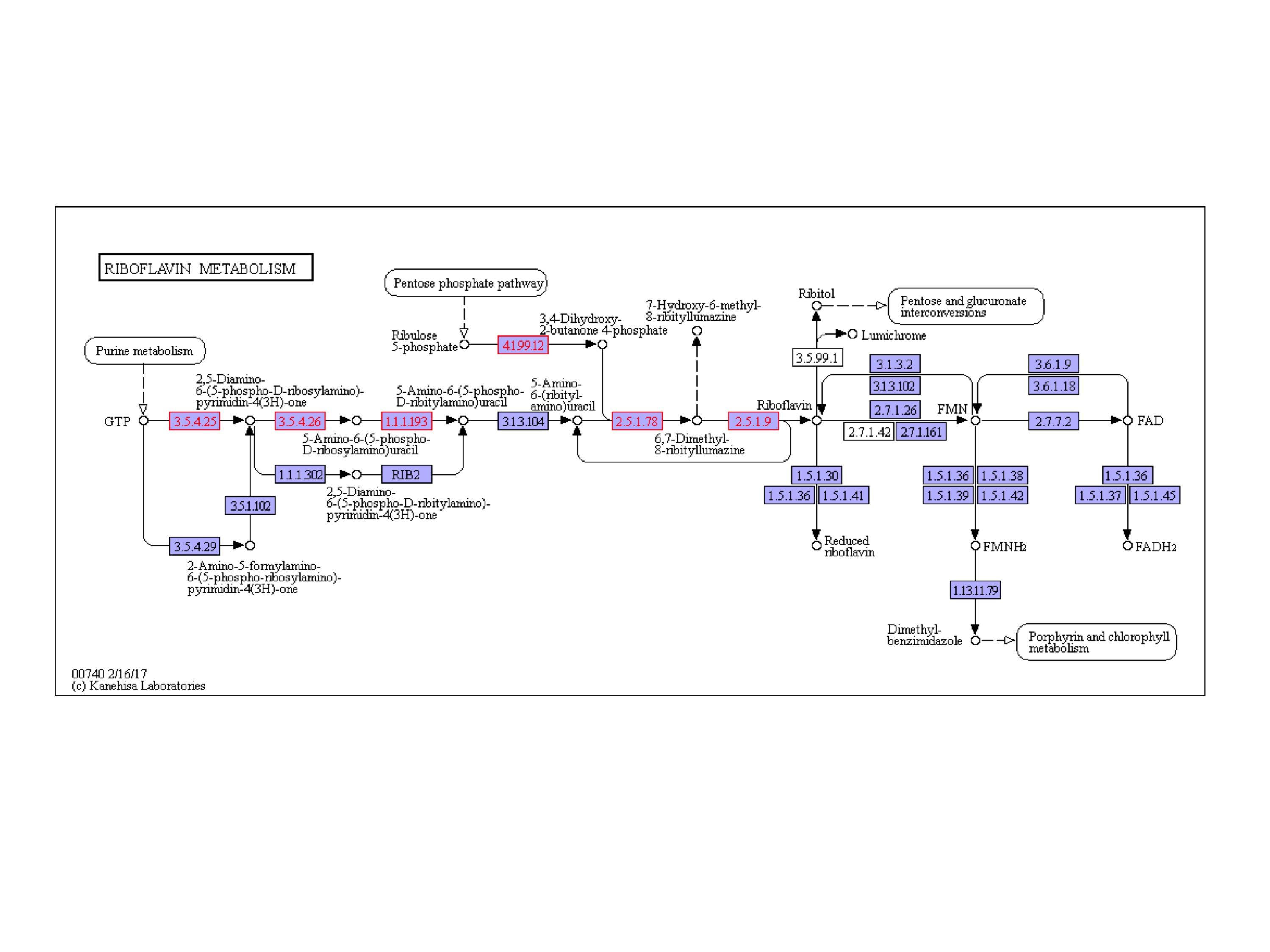

Supplement: FIGURE S3 — Overview of riboflavin metabolism of L. nagelii TMW 1.1827 generated in KEGG mapper. The EC numbers colored in red show up-regulated enzymes of L. nagelii in the presence of S. cerevisiae. [file Image_3.JPEG]
