# Supplementary material for: Proteomic Analysis of Lactobacillus nagelii in the Presence of Saccharomyces cerevisiae Isolated From Water Kefir and Comparison With Lactobacillus hordei
Source: Front Microbiol. 2019 Feb 28;10:325. doi: 10.3389/fmicb.2019.00325 (PMC6413804; doi:10.3389/fmicb.2019.00325)
Supplement: TABLE S2 — List of annotated enzymes by NCBI and RAST involved in carbohydrate metabolism (glycolysis, pentose phosphate pathway, pyruvate metabolism, and TCA cycle). [file Data_Sheet_2.docx]

Table S2. List of annotated enzymes by NCBI and RAST involved in carbohydrate metabolism (glycolysis, pentose phosphate pathway, pyruvate metabolism and TCA cycle).

| Number | Enzyme | EC number | Locus tag from NCBI | RAST-ID |
| --- | --- | --- | --- | --- |
| 1 | Glucokinase | EC 2.7.1.2 | BSQ50_07330 | peg_1459 |
| 2 | Glucose-6-phosphate isomerase | EC 5.3.1.9 | BSQ50_08580 | peg_1678 |
| 3 | 6-phosphofructokinase | EC 2.7.1.11 | BSQ50_06330 | peg_1260 |
| 4 | Fructose-bisphosphate aldolase | EC 4.1.2.13 | BSQ50_04840  BSQ50_07790 | peg_956  peg_1552 |
| 5 | Triose-phosphate isomerase | EC 5.3.1.1 | BSQ50_02645 | peg_510 |
| 6 | Glyceraldehyde 3-phosphate dehydrogenase | EC 1.2.1.12 | BSQ50_02635 | peg_508 |
| 7 | Phosphoglycerate kinase | EC 2.7.2.3 | BSQ50_02640 | peg_509 |
| 8 | Phosphoglycerate mutase | EC 5.4.2.1 | BSQ50_02110  BSQ50_09155 | peg_403  peg_1797 |
| 9 | Enolase | EC 4.2.1.11 | BSQ50_02650  BSQ50_05885 | peg_511  peg_1168 |
| 10 | Pyruvate kinase | EC 2.7.1.40 | BSQ50_06325 | peg_1259 |
| 11 | (L/D) Lactate dehydrogenase | EC 1.1.1.27/28 | BSQ50_01780  BSQ50_01955  BSQ50_05430  BSQ50_06640  BSQ50_08630 | peg_342  peg_379  peg_1074  peg_1321  peg_1688 |
| 12 | Glucose-6-phosphate dehydrogenase | EC 1.1.1.49 | BSQ50_02990 | peg_579 |
| 13 | 6-phosphogluconolactonase | EC 3.1.1.31 | BSQ50_02805 | peg_542 |
| 14 | Phosphogluconate dehydrogenase | EC 1.1.1.44 | BSQ50_06855  BSQ50_10385 | peg_1363  peg_2045 |
| 15 | Ribulose-phosphate 3-epimerase | EC 5.1.3.1 | BSQ50_04865  BSQ50_07245 | peg_961  peg_1441 |
| 16 | Phosphoketolase | EC 4.1.2.9 | BSQ50_09315 | peg_1830 |
| 17 | Acetate kinase | EC 2.7.2.1 | BSQ50_01685  BSQ50_04570  BSQ50_07705  BSQ50_08365  BSQ50_09355 | peg_325  peg_901  peg_1535  peg_1633  peg_1838 |
| 18 | Phosphate acetyltransferase | EC 2.3.1.8 | BSQ50_02695 | peg_520 |
| 19 | Acetaldehyde dehydrogenase | EC 1.2.1.10 | BSQ50_04565 | peg_900 |
| 20 | Alcohol dehydrogenase | EC 1.1.1.1 | BSQ50_00575  BSQ50_04565  BSQ50_05330  BSQ50_09365  BSQ50_09675  BSQ50_09870 | peg_116  peg_900  peg_1053  peg_1840  peg_1902  peg_1943 |
| 21 | Citrate lyase | EC 4.1.3.6 | BSQ50_03225  BSQ50_03230 | peg_625  peg_626 |
| 22 | Oxaloacetate decarboxylase | EC 4.1.1.3 | BSQ50_03205 | peg_621 |
| 23 | Pyruvate oxidase | EC 1.2.3.3 | BSQ50_00690  BSQ50_08185 | peg_141  peg_1616 |
| 24 | Pyruvate dehydrogenase | EC 1.2.4.1 | BSQ50_00245  BSQ50_00250 | peg_49  peg_50 |
| 25 | Pyruvate formate lyase | EC 2.3.1.54 | BSQ50_04390  BSQ50_09420 | peg_863  peg_1851 |
| 26 | Acetolactate synthase | EC 2.2.1.6 | BSQ50_00550  BSQ50_01140 | peg_112  peg_231 |
| 27 | α󠇌-acetolactate decarboxylase | EC 4.1.1.5 | BSQ50_10370 | peg_2042 |
| 28 | Acetoin reductase | EC 1.1.1.4 | BSQ50_05200 | peg_1026 |
| 29 | Ribose-5-phosphate isomerase | EC 5.3.1.6 | BSQ50_02120  BSQ50_04870  BSQ50_08850  BSQ50_09890 | peg_405  peg_962  peg_1732  peg_1947 |
| 30 | Transketolase | EC 2.2.1.1 | BSQ50_03545 | peg_688 |
