# Supplementary material for: Proteomic Analysis of Lactobacillus nagelii in the Presence of Saccharomyces cerevisiae Isolated From Water Kefir and Comparison With Lactobacillus hordei
Source: Front Microbiol. 2019 Feb 28;10:325. doi: 10.3389/fmicb.2019.00325 (PMC6413804; doi:10.3389/fmicb.2019.00325)
Supplement: TABLE S3 — List of enzymes involved in fatty acid biosynthesis. [file Table_3.docx]

Table S3. List of enzymes involved in fatty acid biosynthesis.

| Number | Enzyme | EC number | Locus tag from NCBI | RAST-ID | Proteome |
| --- | --- | --- | --- | --- | --- |
| 1 | Acetyl-CoA carboxylase | EC 6.4.1.2 | BSQ50_02960 BSQ50_02970  BSQ50_02975  BSQ50_02980 | peg_573  peg_575  peg_576  peg_577 | +  +  +  + |
| 2 | Beta-hydroxyacyl-acyl-carrier-protein dehydratase (FabZ) | EC 4.2.1.59 | BSQ50_02965 | peg_574 | + |
| 3 | 3-oxoacyl-acyl-carrier-protein synthase II (FabF) | EC 2.3.1.179 | BSQ50_02955 | peg_572 | - |
| 4 | Malonyl CoA-acyl carrier protein transacylase (FabD) | EC 2.3.1.39 | BSQ50_02945 | peg_570 | + |
| 5 | 3-oxoacyl-acyl-carrier protein reductase (FabG) | EC 1.1.1.100 | BSQ50_00780  BSQ50_02855  BSQ50_02950 | peg_159  peg_552  peg_571 | +  +  + |
| 6 | 3-oxoacyl-acyl-carrier-protein synthase III (FabH/FabY) | EC 2.3.1.180 | BSQ50_02090  BSQ50_02935 | peg_399  peg_568 | +  + |
| 7 | Enoyl-acyl-carrier-protein reductase (FabI/FabK/ FabV) | EC 1.3.1.9 | BSQ50_02985 | peg_578 | + |

Note: “+” presents, that this enzyme annotated in the genome is also identified in proteome, “-” presents, that this enzyme annotated in the genome is not identified in proteome.
